# Food Bank–Based Diabetes Prevention Intervention to Address Food Security, Dietary Intake, and Physical Activity in a Food-Insecure Cohort at High Risk for Diabetes

**DOI:** 10.5888/pcd17.190210

**Published:** 2020-01-09

**Authors:** Kate Cheyne, Morgan Smith, Elizabeth M. Felter, Martha Orozco, Eric A. Steiner, Yuae Park, Tiffany L. Gary-Webb

**Affiliations:** 1 Alameda County Community Food Bank, Oakland, California; 2 Feeding America, Chicago, Illinois; 3 Department of Behavioral and Community Health Sciences, University of Pittsburgh Graduate School of Public Health, Pittsburgh, Pennsylvania; 4 Department of Epidemiology, University of Pittsburgh Graduate School of Public Health, Pittsburgh, Pennsylvania

## Abstract

**Purpose and Objectives:**

Although food insecurity is associated with poor dietary intake and risk of chronic disease, few studies have demonstrated the effectiveness of diabetes prevention interventions delivered through food banks. Food banks serve vulnerable communities. The purpose of this pilot project was to assess the effectiveness of a food bank–delivered intervention aimed at improving food security and reducing risk factors for type 2 diabetes among at-risk clients.

**Intervention Approach:**

We screened adult English- and Spanish-speaking food bank clients for type 2 diabetes risk at 12 community food distribution sites in Alameda County, California. Screening and enrollment for a pilot intervention took place from November 2017 to March 2018. Intervention components were delivered from November 2017 through March 2019. The intervention included monthly diabetes-appropriate food packages, text-based health education, and referrals to health care.

**Evaluation Methods:**

Food bank staff members administered surveys to participants at baseline, 6 months (midpoint), and 12 months (postintervention); participants self-reported all responses. Primary outcomes assessed were food security status, dietary intake, health-related behaviors, and body mass index (BMI). Information on demographic characteristics, food pantry access, health care use, and symptoms of depression was also collected.

**Results:**

We screened 462 food bank clients for eligibility. Of the 299 who were eligible, 244 enrolled; 90.6% were female, 80.1% were Hispanic, and 49.1% had an annual household income less than $20,000. At baseline, 68.8% of participants had low or very low food security. At midpoint, participants had significant improvements in food security status, dietary intake, physical activity, health status, and depression scores. Mean BMI did not change.

**Implications for Public Health:**

This intervention demonstrated that food banks can effectively screen clients at high risk for diabetes and improve household food security and other risk factors for diabetes. Food banks may be an important and strategic partner for health care systems or community-based organizations working to prevent diabetes in food-insecure populations.

**Summary**

**What is already known on this topic?**

Food insecurity is a risk factor for type 2 diabetes, and household food insecurity is more prevalent where a household member has diabetes.

**What is added by this report?**

Food bank clients at risk for diabetes were offered a year-long diabetes prevention pilot intervention near Oakland, California. The intervention featured diabetes-appropriate food, text-based education, and health care referrals. At midpoint (6 months), we found significant improvements in food security, dietary intake, physical activity, and health status. Twelve-month results were unchanged from midpoint.

**What are the implications for public health practice?**

Food banks, which serve highly vulnerable communities, can improve food security and nutrition, but further reducing chronic disease risk and improving health outcomes may require additional partnerships.

## Introduction

Food insecurity is a risk factor for type 2 diabetes (1,2), and household food insecurity is more prevalent when a household member has diabetes (3). The US Department of Agriculture (USDA) defines food insecurity as a lack of consistent access to enough food for an active, healthy life (4). Compared with people in food-secure households, people in food-insecure households are more likely to report poorer health and symptoms of depression (5) and have a higher risk for diet-sensitive chronic conditions such as obesity, hypertension, and diabetes (6). Food-insecure patients with diabetes are also more likely than food-secure patients to have worse diabetes control (eg, hemoglobin A_1c_ levels), potentially increasing risk for diabetes-related complications (7,8). Nationally, food insecurity affects more than 37.2 million people (9) and accounts for $77.5 billion in additional annual health care costs in the United States (10).

Food banks serve a critical need in communities across the country. Feeding America, the nation’s largest domestic hunger relief organization, collaborates with a network of 200 food banks and more than 60,000 affiliated food programs and pantries in the United States to provide food and services to more than 46 million people each year (11).

Food banks may be ideal partners or providers of disease prevention–focused programs for several reasons. First, food banks serve communities that have low socioeconomic status and a high risk for poor health. Nearly 3 of 4 client households in Feeding America’s network are at or below 100% of the federal poverty level (11). Second, food-insecure households accessing a charitable food network may interact with a food bank more frequently than they interact with their health care providers, positioning food banks as venues for delivering health-related interventions to food-insecure populations at high risk for disease. Finally, food provision in vulnerable communities is the expertise of food banks. Food banks can provide nutritionally appropriate foods to food-insecure households that may otherwise struggle to maintain a diet suitable for disease prevention or management.

## Purpose and Objectives

The purpose of this pilot project was to assess the effectiveness of a food bank–delivered intervention aimed at improving food security, dietary intake, and other risk factors for type 2 diabetes among food-insecure clients at risk for diabetes. We assessed the effect of diabetes-appropriate supplemental food and text-based education. Increased national interest in food insecurity among researchers, the public health community, and health care systems during the last decade (12) has produced a growing body of evidence (13–16) showing how food banks can effectively support health promotion and/or management of diet-sensitive chronic diseases such as diabetes. However, to our knowledge, no studies have examined the effectiveness of diabetes prevention strategies in a food bank setting.

## Intervention Approach

Feeding America, in partnership with the University of Pittsburgh, developed the objectives, project framework, and evaluation plan for the pilot intervention. Intervention elements were based on previous food bank programs (13,14) that leveraged food bank capacities and operational expertise to screen clients for disease risk and provide targeted nutrition services. Feeding America selected Alameda County Community Food Bank (ACCFB), in Oakland, California, as the intervention site through a competitive application process that considered 1) previous experience implementing formal research and evaluation protocols with fidelity, 2) capacity to support intervention components, and 3) leadership support. The project received approval from Copernicus Group Institutional Review Board. Screening and enrollment took place on a rolling basis, commencing in November 2017 and concluding in late March 2018. Intervention components were delivered from November 2017 through March 2019. We collected data at baseline, 6 months (midpoint), and 12 months (postintervention).

### Characteristics of intervention setting

ACCFB, located in the heart of the Bay Area in California, serves an estimated 1 in 5 households in a linguistically and culturally diverse geography. ACCFB distributes roughly 35 million pounds of food each year through a network of more than 200 partner agencies and direct service programs. ACCFB was the first food bank in the nation to eliminate the distribution of soda and other sugary beverages in 2005. The food bank formalized a robust nutrition policy in 2012, and since 2014, the food bank has participated in 4 formal research studies. Roughly 60% to 65% of the volume that ACCFB distributes through its partner network is fresh (perishable, noncanned) fruits and vegetables.

### Intervention components

Project intervention activities included monthly distribution of diabetes-appropriate food packages to participants; text-based health promotion education addressing physical activity and nutrition; text-based administrative and engagement messages; and referrals to health care and community-based diabetes prevention programs (DPPs).

**Diabetes-appropriate food packages.** Food packages were designed to increase access to and consumption of foods appropriate for diabetes prevention. Packages were created to approximately mirror USDA’s MyPlate and Choose Healthy Options Program (17). These guidelines emphasize fresh produce, vegetables and fruits, whole grains, lean proteins, low-fat dairy, and healthy fats. Project packages contained shelf-stable products, including lean proteins, legumes, fruits and vegetables, and whole grains. Only canned products that were low in sodium and low in added sugars were included. Study participants received supplemental project-specific food packages, in addition to having access to the food available through normal pantry distributions (ie, including fresh fruits and vegetables, proteins, and dairy).

**Text-based health promotion education.** By January 2018, all participants were enrolled in health promotion education programming delivered through CareMessage (www.caremessage.org) — a nonprofit mobile health technology platform that designs mobile health tools for underserved patient populations. Participants first received a 24-week physical activity module, followed by a 24-week nutrition program. Each module delivered 3 to 5 text messages per week to participants to educate and nudge participants toward healthy behavior modifications. The text-based program was initiated for each participant by food bank staff members and was delivered in the participant’s language of preference. By midpoint, participants had completed the physical activity program and were starting the nutrition module. Physical activity and nutrition education are core content areas of DPPs. Although the text-based health promotion programming was not a substitute for DPP classes, it did provide relevant and complementary health information to participants.

**Text-based engagement and administrative messaging.** All participants who provided consent for text messaging received general administrative messages during the project, with the goal to maintain or increase engagement in activities (primarily picking up project food packages). Messages included distribution reminders, date or time changes, and survey reminders. All messages were sent via the CareMessage platform, managed by food bank staff members, and delivered in the clients’ language of preference.

**Referrals to community-based DPPs.** An initial goal of the project was to connect food bank clients to an existing community-based DPP for education and support aimed at lowering clients’ diabetes risk. However, the DPP provider that the food bank had planned on working with underwent organizational changes and no longer had capacity to provide DPP classes during the project period. Food bank staff members were not able to find a replacement community-based DPP partner within the first 6 months of the study. Originally, the project was designed so that participants would be offered *either* a referral to a formal community-based DPP *or* the text-based health promotion education programming. In January 2018, we decided to enroll all participants who were interested in a community-based DPP into the text-based health promotion programs. When the project’s enrollment closed in March 2018, we had initiated the text-based health program for 203 participants, including the 110 participants who initially expressed interest in the community-based DPP.

## Evaluation Methods

The evaluation objectives for this pilot project were to assess changes in household food security, weekly minutes of physical activity, consumption of healthy foods (particularly fruits and vegetables), and weight/body mass index (BMI, measured as weight in kilograms divided by height in meters squared [kg/m^2^]). Community-based DPPs that use the curricula available through the Centers for Disease Control and Prevention (CDC) expect the main outcomes to be demonstrated within the first 6 months of the program and maintained in months 6 through 12. In conceptualizing this study, we had originally planned to align the 12 months of supplemental food packages with the duration of a community-based DPP, but we were unable to offer the DPP in the first 6 months. The text-based program we offered is not recognized as a replacement for DPP; DPP’s strength is an intensive peer-learning model that requires active engagement and interaction between participants during the year-long program. For this pilot project, we expected improvements in food security, dietary quality, and physical activity to improve within the first 6 months of the program and that improvements would be maintained in months 6 through 12. We did not expect to see improvement in weight or BMI once we were unable to offer referral to a community-based DPP. We felt these expectations were consistent with the logic of the national DPP and with other research done in conjunction with food banks.

### Data sources

The primary data collection tools for the project were surveys completed at baseline, midpoint, and postintervention, at which point participants exited the program. All survey data, including weight and height, from which we computed BMI values, were self-reported. Participant data were collected by using electronic tablets and a centralized data collection platform (Qualtrics), which was managed by research team members at the University of Pittsburgh. Survey versions were available in English and Spanish and were administered in person by food bank staff members. Food bank staff members timed the midpoint and postintervention surveys by measuring 6 months and 12 months, within a 6-week window, from the participant’s enrollment date. Participants were not required to complete surveys to receive the intervention. Participants received a $10 gift card upon completion of each of the baseline, midpoint, and postintervention surveys as compensation for their time.

### Measures

We used a screening survey to assess diabetes risk and project eligibility and collect data on demographic characteristics. The screening survey included CDC’s 7-item Prediabetes Risk Test (https://www.cdc.gov/prediabetes/takethetest). The baseline, midpoint, and postintervention surveys consisted of questions on food security, dietary intake, use of a food pantry, participation in the Supplemental Nutrition Assistance Program (SNAP), general health, height (at baseline only) and weight, and satisfaction with the project (midpoint and postintervention only). Because a high BMI is a metric used to determine DPP eligibility and weight loss is a goal in the DPP, we collected self-reported data on height and weight. Previous studies demonstrated the ability to assess significant changes in food security and dietary intake during a 6-month nutrition intervention (13,14). As previously stated, we prioritized changes in food security and dietary intake at 6 months and evaluated for maintenance and changes again at 12 months once we were no longer able to provide a DPP referral.

We used the USDA Economic Research Service’s 6-item screener (18) to assess food security status of participant households at baseline, midpoint, and postintervention. A food security score, scaled from 0 to 6, was calculated by summing individual affirmative answers to the 6-item assessment. The higher the score, the greater the food insecurity. Food security status was categorized according to USDA guidelines as very low (5 or 6); low (2–4); and high or marginal (0 or 1). We adapted questions on dietary intake, SNAP participation, and other measures from the FRESH Foods Survey (19) and questions on health status and number of physical activity minutes per week from CDC’s Behavioral Risk Factor Surveillance System (20). We calculated BMI by using participant-reported data on height and weight.

We used the 2-item Patient Health Questionnaire-2 (PHQ-2) to assess symptoms of depression among participants. This questionnaire asks about the frequency of depressed mood in the previous 2 weeks, with a score ranging from 0 to 6; if the score is 3 or greater, a major depressive condition is likely (21).

We also measured participant engagement in the project. We established 2 categories of engagement: a participant receiving at least 70% of program food packages was categorized as highly engaged, and a participant receiving less than 70% was categorized as less engaged.

### Participant recruitment

During project development, we determined a target number of participants (N = 250) by considering operational and programming criteria and reviewing recently conducted programs (13,14) that had similar aims and objectives rather than by considering statistical criteria (ie, ascertaining a sample size appropriate for statistical purposes).

We recruited participants from 12 food pantries affiliated with the food bank through flyers, posters, in-person announcements, and word-of-mouth. Screening and enrollment began in November 2017 and concluded in late March 2018. To identify eligible participants, food bank staff members and volunteers administered a screening questionnaire to adult food pantry clients as they waited in line at food distribution sites. Screening questions assessed eligibility (language, age, diabetes history) and included demographic questions (eg, sex, household income, race, ethnicity). Inclusion criteria were a clinical history of prediabetes (by self-report) or a high score (≥9) on CDC’s Prediabetes Risk Test, existing or new food pantry client, aged 18 or older, and English or Spanish verbal fluency. Exclusion criteria were the following: any previous diagnosis of diabetes (not gestational diabetes), pregnancy, fewer than 6 weeks postpartum, or cognitive impairment. Eligible clients who met inclusion criteria and were interested in participating were considered to be enrolled after providing informed consent and contact information and selecting project activities of interest.

At enrollment and during follow-up assessments, participants were asked about their access to primary health care providers. Participants who stated they did not have a primary care provider were given information about local community health care organizations and encouraged by project staff members to establish contact with a health care clinic.

### Statistical analyses

All participant surveys were completed from November 2017 through May 2019. Research team members from the University of Pittsburgh conducted descriptive (univariate and bivariate) analyses to assess significant changes in participant metrics between enrollment and midpoint and between enrollment and 12 months. We used χ^2^ analyses to describe differences in categorical variables and Fisher exact tests when cell sizes were small (<5 participants). We used *t* tests to describe differences in continuous variables. We considered results to be significant at *P* ≤ .05. All statistical analyses were performed by using Stata version 14 (StataCorp LLC).

## Results

Food bank staff members screened 462 food pantry clients aged 18 or older at 12 sites beginning in November 2017. Of the 422 clients who completed CDC’s Prediabetes Risk Test, 244 (57.8%) were eligible, consented to participate, and completed the baseline survey. Of the 422 clients, we excluded 163 for the following reasons: 123 clients had a score less than 9 on CDC’s Prediabetes Risk Test, 32 clients had a previous diagnosis of diabetes, 6 clients discontinued the screening process, and 2 clients were pregnant or fewer than 6 weeks postpartum (Figure). A total of 244 participants consented to receive project food packages and administrative text messages throughout the year-long project. Additionally, 83.2% (n = 203) were interested in the text-based education at baseline, and 45.1% (n = 110) were interested in a referral to a community-based DPP provider.

**Figure:**
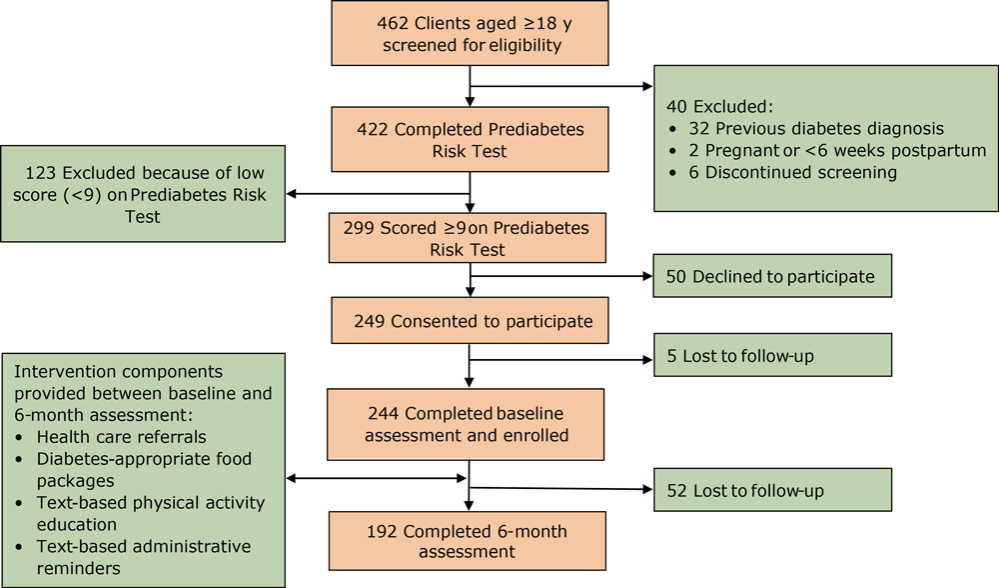
Pilot project enrollment and implementation from baseline to 6 months, food bank–based diabetes prevention intervention, Alameda, California, 2017–2019. The Prediabetes Risk Test is available from the Centers for Disease Control and Prevention at https://www.cdc.gov/prediabetes/takethetest.

### Baseline characteristics of participants

Of 192 participants who completed both the baseline survey and midpoint survey, 174 (90.6%) were female. Of the 186 participants who answered the question on race/ethnicity, 149 (80.1%) were Hispanic or Latino; 14 (7.5%) were black or African American, and 13 (7.0%) were Asian (Table 1). Using self-reported height and weight at baseline, we found that 95.9% of participants were overweight (BMI 25.0–29.9 kg/m^2^) or obese (BMI >30 kg/m^2^). Nearly half (49.1%) of participants reported a household income less than $20,000, and 59.9% reported renting as their living situation.

**Table 1:** Baseline Sociodemographic Characteristics of Participants (N = 192) in a Food Bank–Based Diabetes Prevention Intervention in Alameda County, California, 2017–2019^a^

Characteristic	No. of Respondents	Value
**Age, mean (SD), y**	192	48.5 (12.7)
**Female sex**	192	174 (90.6)
**Race/ethnicity**		
Asian	186	13 (7.0)
Black or African American		14 (7.5)
Hispanic or Latino		149 (80.1)
Other		10 (5.4)
**Body mass index^b^**		
Normal or healthy (18.5–24.9)	192	8 (4.2)
Overweight (25.0–29.9)		66 (34.4)
Obese (≥30.0)		118 (61.5)
**Annual household income, $**		
<20,000	169	83 (49.1)
20,000–39,999		64 (37.9)
40,000–59,999		17 (10.1)
60,000–79,999		5 (3.0)
**Education**		
<High school or GED	192	108 (56.3)
Completed high school or GED		40 (20.8)
Some college, but no degree		13 (6.8)
Completed 2-year degree		13 (6.8)
Completed 4-year degree or higher		18 (9.4)
**Employment status**		
Not employed	192	48 (25.0)
Retired, disabled, homemaker/stay-at-home parent, or student		72 (37.5)
Employed in temporary or part-time job		37 (19.3)
Employed full time		28 (14.6)
Other		7 (3.6)
**Home ownership**		
Rent	192	115 (59.9)
Own		33 (17.2)
Other		44 (22.9)
**SNAP benefits**		
No. of households receiving SNAP at baseline	192	58 (30.2)
No. of households receiving SNAP at 6 months	192	44 (22.9)
No. of households not receiving SNAP at baseline, but receiving it at 6 months	192	12 (6.3)

Abbreviation: SNAP, Supplemental Nutrition Assistance Program.

a All values are number (percentage) unless otherwise noted. Food bank staff members administered surveys to food pantry clients aged ≥18 at 12 sites. Includes only participants who completed both the baseline survey and the midpoint survey and had no missing values. Numbers may not add to 192 because not all participants answered all questions. Percentages are based on the number of participants who answered question; percentages may not sum to 100 because of rounding.

b Calculated as weight in kilograms divided by height in meters squared (kg/m^2^) using self-reported height and weight at baseline.

At baseline, 65.8% (125 of 190) of respondents indicated that “worrying food would run out” was “sometimes true” or “often true”; 71.7% (137 of 191) of respondents indicated that not being able to afford balanced meals was “sometimes true” or “often true”; 43.6% (82 of 188) of respondents indicated that household adults skip meals; 28.9% (13 of 45) of respondents reported household adults skip meals almost every month; 68.8% (132 of 192) of respondents had low or very low food security status; and 30.2% (58 of 192) of respondents were participating in SNAP.

In addition, 84.8% (162 of 191) of respondents reported having a place to go when they are sick and need advice about their health, and 92.1% (176 of 191) of respondents reported having health insurance coverage. Most (79.4%) participants reported last visiting a physician within the past 6 months for a routine checkup. In our study population, 92.1% (176 of 191) of respondents reported having health insurance coverage: of the 106 who answered about type of coverage, 38.7% (n = 41) reported coverage through Medicaid, 23.6% (n = 25) indicated private insurance, and 23.6% (n = 25) reported benefits through Alameda County’s HealthPAC program, which offers health care services to low-income residents not otherwise covered by private insurance or Medicaid. Despite good health care coverage in this population, 19.9% (38 of 191) of respondents reported that they found it difficult to see a physician because of cost.

### Midpoint changes

At midpoint, 192 of 244 participants (78.7%) completed surveys administered by food bank staff members; 52 were lost to follow-up. We found significant improvements in food security status, dietary intake, physical activity, and depression scores. We found no significant changes in weight or BMI.

**Food security status.** The percentage of participants reporting that household adults skip meals decreased from 43.6% at baseline to 29.3% (Pearson χ^2^ = 98.6, *P* < .001) at midpoint (Table 2). The percentage of participants with low or very low food security status decreased from 68.8% at baseline to 62.5% at midpoint (Pearson χ^2^ = 72.6, *P* < .001).

**Table 2:** Changes in Food Security at 6-Month Assessment Among Participants (N = 192) in a Food Bank–Based Diabetes Prevention Intervention in Alameda County, California, 2017–2019^a^

Characteristic	No. of Respondents	Baseline, No. (%)	6-Month Assessment, No. (%)	*P* Value^b^
**Food Security**				
**Worried food will run out**				
Never true	190	65 (34.2)	70 (36.8)	<.001
Sometimes true		98 (51.6)	102 (53.7)	
Often true		27 (14.2)	18 (9.5)	
**Cannot afford balanced meals**				
Never true	191	54 (28.3)	59 (30.9)	<.001
Sometimes true		100 (52.4)	110 (57.6)	
Often true		37 (19.4)	22 (11.5)	
**Adults skip meals**	188	82 (43.6)	55 (29.3)	<.001
**Frequency with which adults skip meals, among those who reported skipping meals**				
Only 1 or 2 months	45	8 (17.8)	6 (13.3)	.44
Some months but not every month		24 (53.3)	28 (62.2)	
Almost every month		13 (28.9)	11 (24.4)	
**Eat less than should**	187	84 (44.9)	55 (29.4)	<.001
**Hungry but didn’t eat**	190	40 (21.1)	33 (17.4)	<.001
Food security score, mean (median [IQR])^c^^,^ d	192	2.8 (2.0 [1.0–5.0])	2.3 (2.0 [1.0–4.0])	<.001
**Food security status^c^**				
High or marginal food security	192	60 (31.3)	72 (37.5)	<.001
Low food security		80 (41.7)	87 (45.3)	
Very low food security		52 (27.1)	33 (17.2)	

**Use of Project Food Packages**				
**How much of supplemental food did the participant and household eat?**				
Little	191	NA	3 (1.6)	NA
Some		NA	26 (13.6)	
Most		NA	72 (37.7)	
All		NA	90 (47.1)	
**How much of supplemental food was thrown out or given away?**				
None	192	NA	134 (69.8)	NA
Little		NA	38 (19.8)	
Some		NA	18 (9.4)	
Most		NA	2 (1.0)	

**Pantry Use Variables**				
**Amount of your household’s food supply from food pantries or food giveaways, among those reporting obtaining food from such sources**				
<1 Week’s worth	155	40 (25.8)	43 (27.7)	.13
1 or 2 Weeks’ worth		97 (62.6)	72 (46.5)	
>2 Weeks’ worth		18 (11.6)	40 (25.8)	
**Visited food pantry in the last 4 weeks, among those who reported visiting food pantry ≥1 time**				
Only once — just today	169	63 (37.3)	74 (43.8)	.003
≥2 Times		106 (62.7)	95 (56.2)	

Abbreviation: IQR, interquartile range; NA, not applicable.

a All values are number (percentage) unless otherwise noted. Food bank staff members administered surveys to food pantry clients aged ≥18 at 12 sites. Includes only participants who completed both the baseline survey and the midpoint survey.

b *P* value determined by Pearson χ^2^ test for categorical indicators and paired *t* test for continuous indicators.

c USDA Economic Research Service’s 6-item screener (18) was used to assess food security score and food security status of participant households. The score was determined by summing individual affirmative answers to the 6-item assessment. Score is scaled from 0 to 6, with higher scores indicating low or very low food security. Food security status was categorized as very low (5 or 6); low (2–4); and high or marginal (0 or 1).

d The difference in mean food security score between baseline and 6-month assessment was −0.5 (SD, 1.5).

**Physical activity and general health.** The minutes of physical activity per week reported increased from 95.6 to 145.1 (paired *t* test = 4.05, *P* < .001) among participants, and the percentage of participants who reported regular physical activity at least once per week increased from 62.5% to 80.7% (Pearson χ^2^ = 21.0, *P* < .001) (Table 3). The percentage of participants who reported their health status as poor or fair declined from 73.9% to 60.1% (Fisher exact = 39.19, *P* < .001).

**Table 3:** Changes in Health Outcomes at 6-Month Assessment Among Participants (N = 192) in a Food Bank–Based Diabetes Prevention Intervention in Alameda County, California, 2017–2019^a^

Characteristic	No. of Respondents	Baseline	6-Month Assessment	Difference, Median (IQR)	*P* Value^b^
**General health**					
Positive (excellent, very good, or good)	188	49 (26.1%)	75 (39.9%)	—c	<.001
Negative (fair or poor)		139 (73.9%)	113 (60.1%)		
**Physical activity**					
Participate in regular activity once per week	192	120 (62.5%)	155 (80.7%)	—c	<.001
Minutes of physical activity in an average week, mean (median [IQR])	192	95.6 (60.0 [6.5 to 145.0])	145.1 (120.0 [55.0 to 210.0])	+49.4 (30.0 [−2.5 to 115.0])	<.001
**Score of ≥3 on PHQ-2^d^**	192	48 (25.0%)	29 (15.1%)	—c	<.001
**Body mass index, mean (median [IQR]), kg/m^2^**					
All	184	32.4 (32.0 [28.3 to 35.2])	32.4 (32.0 [28.3 to 35.0])	0 (0 [−0.9 to 1.0])	.90
Female	167	32.6 (32.1 [28.3 to 35.2])	32.6 (32.1 [28.9 to 35.4])	0 (0 [−1.0 to 1.0])	.90
Male	17	30.6 (29.0 [28.0 to 32.6])	30.6 (29.9 [27.5 to 31.9])	0 (0 [−0.8 to 0.2])	.98

Abbreviations: IQR, interquartile range; PHQ-2, Patient Health Questionnaire-2.

a Food bank staff members administered surveys to food pantry clients aged ≥18 at 12 sites. Includes only participants with no missing values.

b *P* value determined by Pearson χ^2^ test for categorical indicators and paired *t* test for continuous indicators.

c Not calculated.

d The PHQ-2 inquires about the frequency of depressed mood in the previous 2 weeks, with a score ranging from 0 to 6; if the score is ≥3, a major depressive condition is likely (21).

**Dietary intake.** Consumption of the following healthy foods increased significantly: green salad, nonfried vegetables, cooked beans, cooked whole grains, whole-grain foods, and fruits and vegetables (Table 4). Consumption of the following unhealthy foods decreased significantly: any sweetened drinks, fried potatoes, candy/chocolate, and cookies/cakes and other sugary dessert foods.

**Table 4:** Changes in Diet-Related Outcomes at 6-Month Assessment Among Participants (N = 192) in a Food Bank–Based Diabetes Prevention Intervention in Alameda County, California, 2017–2019^a^

Outcome	No. of Respondents	Baseline	6-Month Assessment	Difference	*P* Value^b^
**Dietary intake^c^**					
Any sweetened drinks	192	0.60 (0.29 [0.29 to 1.00])	0.41 (0.29 [0 to 0.29])	−0.19 (0 [−.042 to 0])	.002
100% pure fruit juice	192	0.260.29 [0 to 0.29])	0.28 (0.29 [0 to 0.29])	+0.02 (0 [−0.15 to 0])	.56
Water	192	2.45 (3.00 [2.00 to 3.00])	2.57 (3.00 [2.00 to 3.00])	+0.12 (0 [0 to 0])	.07
Fruit	192	0.89 (1.00 [0.29 to 1.00])	0.99 (1.00 [0.29 to 1.00])	+0.10 (0 [−0.15 to 0.71])	.14
Green salad	192	0.43 (0.29 [0.29 to 0.29])	0.52 (0.29 [0.29 to 0.71])	+0.09 (0 [0 to 0.29])	.003
Fried potatoes	191	0.17 (0.29 [0 to 0.29])	0.14 (0 [0 to 0.29])	−0.03 (0 [−0.29 to 0])	.02
Other nonfried potatoes	191	0.24 (0.29 [0 to 0.29])	0.25 (0.29 [0 to 0.29])	+0.01 (0 [0 to 0])	.74
Nonfried vegetables	192	0.48 (0.29 [0.29 to 0.71])	0.56 (0.29 [0.29 to 0.71])	+0.08 (0 [0 to 0.29])	.03
Cooked beans	192	0.54 (0.29 [0.29 to 1.00])	0.62 (0.29 [0.29 to 1.00])	+0.08 (0 [0 to 0.29])	.03
Pizza	192	0.10 (0 [0 to 0.29])	0.12 (0 [0 to 0.29])	+0.02 (0 [0 to 0])	.21
Whole-grain bread	191	0.45 (0.29 [0.29 to 0.71])	0.52 (0.29 [0.29 to 1.00])	+0.07 (0 [0 to 0.29])	.08
Cooked whole grains	192	0.35 (0.29 [0 to 0.29])	0.48 (0.29 [0.29 to 0.71])	+0.13 (0 [0 to 0.29])	<.001
Candy/chocolate	192	0.35 (0.29 [0 to 0.29])	0.23 (0.29 [0 to 0.29])	−0.12 (0 [−0.29 to 0])	<.001
Frozen dessert	192	0.13 (0 [0 to 0.29])	0.16 (0 [0 to 0.29])	+0.03 (0 [0 to 0])	.18
Cookies, cakes, other sugary dessert foods	192	0.32 (0.29 [0 to 0.29])	0.23 (0.29 [0 to 0.29])	−0.09 (0 [−0.29 to 0])	.002
Sugary cereals	191	0.12 (0 [0 to 0.29])	0.11 (0 [0 to 0.29])	0 (0 [0 to 0])	.84
Nonsugary cereals	192	0.20 (0.15 [0 to 0.29])	0.21 (0.29 [0 to 0.29])	+0.01 (0 [0 to 0])	.57
Whole-grain foods	191	0.99 (0.87 [0.58 to 1.29])	1.20 (1.00 [0.58 to 1.58])	+0.21 (0.29 [−0.29 to 0.71])	.001
Fruits and vegetables	191	2.83 (2.58 [1.87 to 3.87])	3.20 (2.87 [2.16 to 4.08])	+0.37 (−0.42 [−0.58 to 1.13])	<.001
**Confident in ability to eat fruits and vegetables every day, no. (%)**					
Agree	188	162 (86.2)	177 (94.2)	NA	.09
Neither agree nor disagree		7 (3.7)	2 (1.1)		
Disagree		19 (10.1)	9 (4.8)		

Abbreviation: IQR, interquartile range; NA, not applicable.

a All values are mean [median (IQR)] daily intake frequency unless otherwise noted. Food bank staff members administered surveys to food pantry clients aged ≥18 at 12 sites. Includes only participants with no missing values. Percentages may not sum to 100 due to rounding.

b *P* value determined by paired *t* test for dietary recall and Fisher exact test for confidence.

c Questions adapted from the FRESH Foods Survey (19).

**Engagement.** Half of participants were categorized as highly engaged and half were less engaged. Regardless of how many packages a participant picked up, food security and fruit and vegetable consumption improved significantly and in similar magnitudes. Highly engaged participants were more food secure than the less engaged group at baseline (Table 5). Although food security improved significantly in both groups, the food security scores of the less engaged group at midpoint were lower than the food security scores of the highly engaged group at baseline.

**Table 5:** Changes in Main Outcomes at 6-Month Assessment for Highly Engaged Participants (n = 95) and Less Engaged Participants (n = 94) in a Food Bank–Based Diabetes Prevention Intervention in Alameda County, California, 2017–2019^a^

Outcome	No. of Respondents	Baseline	6-Month Assessment	Difference	*P* Value^b^
**Engagement, no (%)**					
Highly engaged	189	NA	94 (49.7%)	NA	NA
Less engaged		NA	95 (50.3%)	NA	NA
**Food security score^c^**					
Highly engaged	95	2.47 (2.0 [1.00 to 4.00])	2.03 (2.0 [1.0 to 3.0])	−0.44 (0 [−1.0 to 1.0])	.005
Less engaged	94	3.14 (3.0 [2.00 to 5.00])	2.65 (2.0 [1.0 to 4.0])	−0.49 (0 [−1.0 to 0])	.003
**Fruits and vegetables score^d^**					
Highly engaged	95	2.95 (2.58 [1.87 to 3.87])	3.31 (3.15 [2.16 to 4.16])	+0.36 (0.42 [−0.71 to 1.13])	.02
Less engaged	94	2.72 (2.52 [1.87 to 3.71])	3.10 (2.87 [2.16 to 3.87])	+0.38 (0.29 [−0.42 to 1.13])	.01
**Whole-grain foods score^d^**					
Highly engaged	95	1.02 (0.87 [0.29 to 1.58])	1.28 (1.00 [0.87 to 1.58])	+0.26 (0.29 [−0.29 to 0.71])	.10
Less engaged	94	0.97 (0.87 [0.58 to 1.29])	1.10 (0.87 [0.58 to 1.58])	+0.13 (0 [−0.29 to 0.58])	.01
**Minutes of physical activity in an average week**					
Highly engaged	95	106.8 (60.0 [7.0 to 150.0])	151.5 (120.0 [60.0 to 210.0])	+44.7 (30.0 [0 to 120.0])	.02
Less engaged	94	84.5 (60.0 [0 to 140.0])	138.0 (105.0 [50.0 to 200.0])	+53.5 (30.0 [−3.0 to 100.0])	<.001

Abbreviations: IQR, interquartile range; NA, not applicable.

a Dietary values are mean [median (IQR)] daily intake frequency unless otherwise noted. Food bank staff members administered surveys to food pantry clients aged ≥18 at 12 sites. Highly engaged meant picking up ≥70% of program food packages. Less engaged meant picking up <70% of program food packages.

b *P* value determined by paired *t* test.

c USDA Economic Research Service’s 6-item screener (18) was used to assess food security score and food security status of participant households. The score was determined by summing individual affirmative answers to the 6-item assessment. Score is scaled from 0 to 6, with higher scores indicating low or very low food security. Food security status was categorized as very low (5 or 6); low (2–4); and high or marginal (0 or 1).

d Questions adapted from the FRESH Foods Survey (19).

Consumption of fruits and vegetables between baseline and midpoint improved significantly in both groups. The 2 groups also had similar consumption patterns at baseline and at midpoint. The consumption of whole grains improved significantly in the highly engaged group. We found a similar improvement in the less engaged group, but it was not significant.

### Preliminary analyses at postintervention

At postintervention, 159 of 244 (65.2%) participants completed surveys administered by food bank staff members; 33 participants were lost to follow-up between midpoint and postintervention. We conducted preliminary analyses comparing baseline and postintervention outcomes. Results indicated improved food security, dietary intake, physical activity, health status, and depression scores (*P* < .001 for each). Although results at postintervention remained significantly different from baseline, they were generally unchanged from the results observed at midpoint, indicating maintenance of improvements observed at the 6-month mark.

## Implications for Public Health Practice

The purpose of this pilot project was to assess the effectiveness of a food bank–delivered intervention aimed at improving food security and reducing risk factors for type 2 diabetes among at-risk clients. The screening and enrollment process showed that 7 in 10 clients were at high risk for diabetes. In the participant group, 95.8% were overweight or obese, 43.6% reported adults skipping meals in the household, and nearly 3 in 4 (73.9%) characterized their general health as fair or poor. After a 6-month intervention composed mainly of supplemental diabetes-appropriate food and text-based health education, scores for food security, dietary intake, physical activity, health status, and depression improved significantly. When stratified by engagement level, our results aligned with the results of other studies finding lower levels of participation among people with lower levels of food security (22). This finding may indicate that people with very low food security may have additional barriers that impede engagement. Gains in food security, dietary intake, physical activity, health status, and depression scores demonstrated at postintervention were unchanged from those at midpoint.

BMI did not change significantly from baseline to midpoint or postintervention, but we did not anticipate improvement in BMI because we were not able to offer referrals to a community-based DPP. Improvements in food security and dietary intake outcomes are consistent with improvements found in other food bank–based interventions that focused on diabetes management rather than diabetes prevention (12,13). The retention rate for our project was nearly 80% at midpoint, suggesting that the program was accessible and relevant to a population that faced numerous challenges to program participation (eg, transportation, childcare, work) during the year-long intervention.

In our study population, 92.1% reported having health insurance coverage, which is consistent with the countywide average for health insurance coverage since implementation of the Affordable Care Act (23).

Although we did not explicitly explore the effect of the intervention on dietary intake among nonparticipating household members, participants informally commented to food bank staff members that the intervention benefitted the entire household, including children and other adult household members who may have been struggling to manage diagnosed type 2 diabetes. The rate of participation among women in this project was high. It mirrors the representation of women at the food bank’s distributions generally and rates among women observed in other food bank–based research projects (13,14). Mothers and women often act as nutritional gatekeepers for the household; women are and should be an important group to target for similar programs.

The rate of participation in SNAP among project participants was lower than expected, and it remained relatively unchanged during the intervention. Some households lost their SNAP benefits during the first 6 months of the intervention, while other households gained access to SNAP during this time. Although food bank staff members regularly conduct outreach activities at many distribution sites, not every distribution site for this project had an outreach staff member in attendance to encourage and facilitate SNAP enrollment and maintenance.

This pilot project demonstrated that food banks serve vulnerable populations at high risk for poor health and chronic disease, and that delivery of disease prevention programs through food banks can be effective, accessible, and relevant for populations that may not be able to access similar services through traditional health care systems or community-based programs. Participants in this pilot project had significant improvements in health-related metrics despite not having access to a formal DPP. More work is needed, however, to build DPP infrastructure and the systems necessary to link DPPs to health care providers and community organizations like food banks to ensure high-risk populations can access comprehensive prevention services.

This project had several limitations. The project was offered only in English and Spanish, which limited our ability to screen and enroll all clients at high risk of diabetes. We recruited a convenience sample, and recruitment was driven by the agencies and locations willing to work with food bank staff members. The study was conducted in cooperation with a single food bank in an urban environment and lacked a comparison group, and results may not be generalizable to other populations and communities in the United States. This program was available to participants for only 12 months. Considering the structural challenges and barriers that food pantry clients may face in accessing and maintaining a diet appropriate for diabetes prevention, 12 months may not be sufficient to achieve weight-related outcomes or understand long-term effects. Relatedly, temporal bias may have affected the accuracy of self-reported baseline and follow-up measures.

Social desirability bias may have influenced self-reported risk factors at baseline and outcomes at follow-up. Baseline demographic information aligns with information in previous Hunger in America studies that describe the populations served by food banks. Nearly half of participants in this pilot program were living on less than $20,000 in annual household income. The 2014 Hunger in America study (11) showed that half of ACCFB’s clients get all or most of their food from the food bank.

Lastly, the food bank was not able to implement all intervention components as originally planned. The inability to offer a referral to a community-based DPP may have affected participant engagement and outcomes for those who would have preferred an in-person option. The text-based health education component was also somewhat delayed for this group, which originally requested referrals to community-based DPPs. We made the decision to enroll all participants in the text-based program in January 2018. For some participants, this enrollment was 2 months after they had enrolled in the overall program and began receiving supplemental food packages. Despite this limitation, we did not observe lower rates of program engagement for this group.

Food bank clients are interested in receiving healthy (or healthier) foods from their pantry or food bank (24). Increasingly, many food banks no longer see themselves solely as antihunger organizations but as partners to clients and communities in helping reduce the health concerns that disproportionately affect the communities they serve (25). One in 3 adults nationally are estimated to have prediabetes, and 90% of these adults do not know they have prediabetes (26). In a 2016 study, 46% of all adults in California were estimated to have prediabetes or undiagnosed diabetes (27). With diabetes diagnoses come higher out-of-pocket medical costs (28), and these costs add to the challenges among food-insecure populations in accessing a diabetes-appropriate diet. In this project, we found that 70.9% (299 of 422) of clients screened had prediabetes, according to CDC’s Prediabetes Risk Test, highlighting the opportunity to engage food banks as partners or foci for interventions in populations at high risk of diabetes. More research is needed to identify how and if including a DPP as part of a food bank intervention supports clients in meeting weight-loss goals that are part of diabetes prevention strategies. However, it is imperative that public health and health care systems develop the infrastructure for broad dissemination of evidenced-based programs like the DPP and ensure it is accessible to and tailored for those communities at the highest risk of diabetes.

To achieve the “triple aim” in health care (improved population health, improved patient experience, and reduced health care costs) (29), health care organizations are increasingly working to address social determinants of health — such as food insecurity — with community partners outside traditional health care settings. This work has contributed to an increasing national interest in exploring food banks and food pantries as settings through which health care interventions in general, and diabetes prevention interventions in particular, can be conducted. Further exploration and evaluation of similar models should be pursued, particularly because these models often reach marginalized populations who are at the highest risk for poor health and who face multiple barriers to accessing and using health care services that target disease prevention and health promotion.
